# Sonographic Features of Juvenile Fibroadenoma in Children-a Retrospective Study

**DOI:** 10.2174/0115734056339987250116102211

**Published:** 2025-01-27

**Authors:** Jian Shi, Luzeng Chen, Jingming Ye, Shuang Zhang, Hong Zhang, Yuhong Shao, Xiuming Sun

**Affiliations:** 1 Department of Ultrasound, Peking University First Hospital, Beijing 100034, China; 2 Breast Disease Center, Peking University First Hospital, Beijing 100034, China; 3 Department of Pathology, Peking University First Hospital, Beijing 100034, China

**Keywords:** Juvenile fibroadenoma, Children, Sonographic feature, Low frequency transducer, Posterior acoustic enhancement

## Abstract

**Aims:**

Studies specifically examining the sonographic features of juvenile fibroadenoma in the pediatric population have not been documented. We aimed to analyze sonograms of juvenile fibroadenoma in children.

**Subjects and Methods:**

Patients aged ≤ 18 years who underwent breast ultrasound examinations at our department and had pathologically proven juvenile fibroadenoma from September 2002 to January 2022 were included in this study. Demographic data, clinical findings, and sonograms were retrospectively analyzed. Patients were further divided into the puberty and post-puberty subgroups, and their results were compared.

**Results:**

A total of 24 girls aged 10-18 years with 27 masses diagnosed as juvenile fibroadenomas were identified. The diameter of the masses averaged 5.8 ± 3.3 cm, with a range of 1.5-13.6 cm. Twenty-one (87.5%) patients had a single mass and 3 had double lesions. Over 80% of the lesions were oval-shaped and encapsulated with circumscribed margins and parallel orientation. All masses showed internal hypoechogenicity, either uniform or heterogeneous. For masses that had a diameter > 5 cm, screening with high-frequency transducers revealed no posterior acoustic features or posterior shadowing. However, these features changed to posterior acoustic enhancement when the masses were re-evaluated using low-frequency transducers. Ultrasonic color Doppler showed blood flow in 24 (88.9%) masses. There were no significant differences in the incidence and sonographic features between the two subgroups.

**Conclusion:**

Most juvenile fibroadenomas in children are oval, circumscribed, encapsulated masses with detectable blood flow. All juvenile fibroadenomas presented in this study exhibit internal hypoechogenicity with no posterior acoustic shadowing detected in any cases. Our findings suggest that screening with low-frequency transducers should be performed for a mass that has a diameter > 5 cm.

## 1 INTRODUCTION

Breast fibroadenoma is a type of benign tumor commonly found to be asymptomatic [1, 2]. It is classified into simple and complex fibroadenoma, with the former being characterized by hyperplastic lobules, and the latter exhibiting one or more of the following additional pathological changes: epithelial calcifications, cysts larger than 3 mm, sclerosing adenosis, and papillary apocrine metaplasia [3-5]. Complex fibroadenoma has been reported to occur mostly in women aged over 18 years [6].

Juvenile fibroadenoma, a variant of fibroadenoma, has fewer lobular components than simple fibroadenoma but exhibits both stromal and epithelial hypercellularities [5]. It often presents as a rapidly growing mass mostly seen in children aged 10-18 years, although its occurrence in adults has also been reported [7]. The incidence of fibroadenoma in the pediatric population is relatively low and juvenile fibroadenoma is even rarer [8, 9]. As the developing mammary glands in children are highly sensitive to ionizing radiation, ultrasound (US) is the mainstay of diagnosis for juvenile fibroadenoma. A few case reports have described US examinations of juvenile fibroadenoma in children [10-12]. To date, there is only one case series study that investigated sonographic appearances of juvenile fibroadenoma in 23 patients; however, this study included both children and adults [7]. Studies specifically examining the sonographic features of juvenile fibroadenoma in children and adolescents have not been documented. In this study, we sought to analyze sonograms of juvenile fibroadenoma in patients aged ≤ 18 years.

## 2 SUBJECTS AND METHODS

This study was approved by the Medical Research Review Board of Peking University First Hospital (Approval# 2022-Research-376-001), in compliance with the Declaration of Helsinki, the Code of Ethics of the World Medical Association. Informed consent was waived by the Review Board due to the retrospective nature of the study.

Patients aged ≤ 18 years who underwent breast US examinations and had pathologically proven juvenile fibroadenoma from September 2002 to January 2022 were included in this study. Demographic data, clinical findings, and sonograms were retrospectively retrieved and analyzed.

Gray-scale and color Doppler US examinations were performed according to the protocols established in our department. High-frequency linear transducers ranging from 7-14 MHz were used. For masses with diameters > 5 cm, low frequency curvilinear transducers (i8CX1, 1.8-6.2 MHz, Canon Medical Systems or 6C1HD, 1.5-6.0 MHz, Siemens Medical Solutions) were used to re-evaluate the lesion. Grayscale sonograms were analyzed for mass size and shape, internal echogenicity, lesion boundary and orientation, posterior echo features, presence of calcifications, and ectasia of surrounding ducts using our standard US image-viewing workstation. The blood flow resistance index (RI) in the lesion was recorded by color Doppler. Ultrasonic color Doppler images were assessed for the blood flow in the lesion. The blood flow was graded according to the Alder classification as follows: grade 0 refers to no detectable blood flow; grade 1 is assigned when 1 or 2 small blood vessels with a diameter of < 1 mm are visualized; grade 2 is assigned when 3 or 4 small blood vessels are detected; and grade 3 is assigned when more than 4 blood vessels or an intertwined network of blood vessels are detected [13]. All lesions were categorized according to the US-Based Classification of The American College of Radiology Breast Imaging Reporting and Data System (BI-RADS) [14]. There are significant physical, hormonal, and psychological differences between children in puberty and those post-puberty [15], which may result in different outcomes in these two groups of children in the present study. In view of this and according to current pubertal development in Chinese children [16], we divided all patients into the puberty (10-13 years) and post-puberty (> 13 but ≤ 18 years) subgroups and compared their results.

Data were presented as mean ± standard deviation if they were normally distributed or median (first quartile, the third quartile) if they were not normally distributed. Continuous variables between the two subgroups were analyzed with the two-tailed Student’s t-test. Categorical data between the two subgroups were analyzed with Fisher's exact test. All statistical analyses were performed using GraphPad 8.0 (GraphPad Software, Inc., Boston, USA). A p-value < 0.05 was considered statistically significant.

## 3 RESULTS

Twenty-four female patients aged 10-18 years (median age: 13 years), were included in this study. A total of 27 masses were surgically removed and pathologically proven to be juvenile fibroadenomas postoperatively, with 17 located in the right breast and 10 in the left. Demographic information and clinical data of all patients are shown in Table **1**. Majority of patients (79.2%) had menarche; 21 patients had a single lesion while 3 had double lesions; local pain was reported in 3 patients, two of whom had double masses; axillary lymph node enlargement was found in one case (Table **1**).

The diameter of the masses ranged from 1.5-13.6 cm (5.8 ± 3.3 cm). The sonographic findings are shown in Table **2**. Most lesions were oval, encapsulated, and had circumscribed margins with parallel orientation. All masses exhibited internal hypoechogenicity, which was either uniform or heterogeneous. High-frequency linear transducers used in screening revealed posterior acoustic enhancement in 6 masses, posterior shadowing in 3, and no posterior acoustic features in 18. Five masses (3 with posterior shadowing and 2 with no posterior acoustic features) that had a diameter > 5 cm were re-examined with low frequency transducers. This examination showed that all 5 masses had posterior acoustic enhancement (representative sonograms of a patient are shown in Fig. (**1**). Ultimately, 11 masses (40.7%) displayed posterior acoustic enhancement, while 16 (59.3%) demonstrated no posterior acoustic features (Table **2**). Skin thickening associated with lesions was observed in 12 masses. Calcification and ectasia of surrounding ducts were not detected.

Vascularity in the masses was assessed using ultrasonic color Doppler, and the results are shown in Table **3**. Blood flow was detected in 24 (88.9%) of the 27 masses. A single mass had a blood flow RI > 0.7. According to the BI-RADS system, the masses were classified as follows: 17 as category 3, 8 as category 4a, and 2 as category 4b.

Of the patients studied, 15 were in puberty and 9 were post-pubertal (p = 0.15). The puberty subgroup had 17 masses while the post-puberty subgroup had 10 masses (p = 0.10). The average diameters of the masses were 6.4±3.5 cm and 4.9 ± 3.0 cm for children in puberty and those post-puberty, respectively (p = 0.26). There were no significant differences in the parameters of masses between the two subgroups (all p values > 0.05).

## 4 DISCUSSION

Ultrasound remains a reliable pediatric-specific breast imaging strategy [17]. In the present study, we investigated the sonographic characteristics of juvenile fibroadenomas in 24 girls and reported the following findings: 1) in most cases, juvenile fibroadenoma occurred as a unilateral breast mass; 2) most juvenile fibroadenomas are oval, encapsulated, and circumscribed; 3) all juvenile fibroadenomas exhibited internal hypoechogenicity; 4) posterior acoustic characteristics included posterior acoustic enhancement or no posterior acoustic features; and 5) blood flow was frequently detected in juvenile fibroadenomas.

We observed that 85.2% of juvenile fibroadenomas were oval. A study of 23 women with juvenile fibroadenomas by Kim et al. revealed that 29 out of 34 (85.3%) masses appeared oval [7], which aligns with our finding, although Kim et al. included adults with an average age of 25 years [7]. A circumscribed margin was seen in 26 masses (96.3%) in our series, which is higher than the 70.6% (24/34) reported by Kim et al. [7]. This discrepancy is probably due to the age difference in patients (an average age of 25 years reported by Kim et al. vs an average age of 13 years in our study) or the limited sample size of the study. The shapes and margins of juvenile fibroadenomas we reported in this study are similar to those of simple fibroadenomas in children described by Sanchez et al. [18].

All masses in our patients showed uniform or heterogeneous internal hypoechogenicity. This finding contrasts with the report by Kim et al., which demonstrated that approximately half of the 34 juvenile fibroadenomas exhibited internal hypoechogenicity, while the remaining half displayed isoechogenicity [7]. The reason for these different observations remains unknown. Screening with high-frequency transducers generated different posterior acoustic patterns in our series, including posterior acoustic enhancement, posterior shadowing, and no posterior acoustic features. Notably, when high-frequency transducers were employed, masses with a diameter greater than 5 cm exhibited posterior shadowing or no posterior acoustic features in our patients. However, upon re-examination with low-frequency transducers, these masses demonstrated a transition from posterior shadowing or no posterior acoustic features to posterior acoustic enhancement. Interestingly, a study examining US imaging of 44 simple fibroadenomas in children reported posterior acoustic enhancement in 43 masses and posterior shadowing in only one lesion [18]. Another study, which focused on 32 juvenile fibroadenomas in female patients, including both children and adults, found posterior acoustic enhancement in 22 masses, posterior shadowing in 1 mass, and no posterior acoustic features in 9 masses [7]. Notably, both studies utilized high-frequency linear transducers (7-14 MHz) but did not provide information on the size of the masses exhibiting posterior shadowing [7, 18]. In a case report, Giannos et al. described the US features of a juvenile fibroadenoma with a diameter of 13 cm in a 12-year-old girl [12]. Using a low-frequency transducer (4-9 MHz), they found that the tumor exhibited posterior acoustic enhancement [12]. Based on these literature reports and our findings, we believe it is crucial to consider tumor size when posterior shadowing or no posterior acoustic features are detected using high-frequency transducers. Low-frequency transducers should be employed to better characterize the posterior acoustic features of large juvenile fibroadenomas.

We did not observe calcification in any of the lesions. In contrast, Kim et al. reported calcification in 3 out of 34 juvenile fibroadenomas in female patients, which included adults, with 3 subjects aged over 40 years [7]. However, these authors did not elaborate on whether calcification occurred in children or adults [7]. In another study, Sanchez et al. did not detect calcification in 44 simple fibroadenomas in children [18], which is in agreement with our results. The association between calcification in juvenile fibroadenomas and age remains unclear. Blood flow was frequently detected in our cases, similar to previous reports [7].

Given the significant physical, hormonal, and psychological differences between children in puberty and those post-puberty [15], we compared results between these two subgroups. Our analysis revealed no significant differences in the incidence and sonographic features. However, this study has the following limitations: 1) it is a retrospective study; 2) the sample size is limited; and 3) all patients were drawn from a single center. These factors may affect the generalizability of our findings.

## CONCLUSION

The vast majority of juvenile fibroadenomas in children are oval, circumscribed, and encapsulated masses with detectable blood flow. All juvenile fibroadenomas presented in this study exhibited internal hypoechogenicity with no posterior acoustic shadowing detected in any cases. There were no significant differences in the incidence and sonographic features between children in puberty and those post puberty. Our findings suggest that screening with low-frequency transducers should be performed for a mass with a diameter > 5 cm, which may have an implication for clinical practice in this field.

## Figures and Tables

**Fig. (1):**
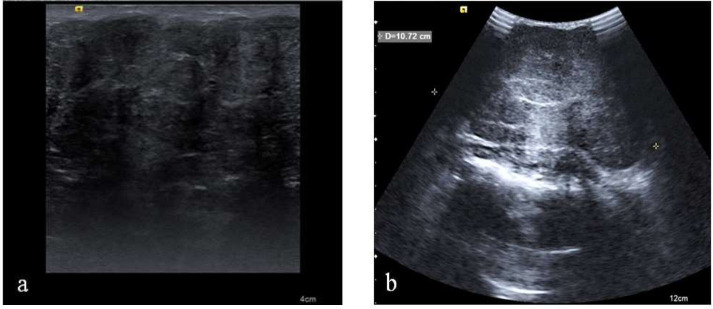
A mass with an 8 cm diameter examined by a high frequency transducer (**a**) and a low frequency transducer (**b**). The high frequency transducer gave rise to posterior shadowing (**a**) that changed to posterior acoustic enhancement when the mass was re-evaluated using a low frequency transducer (**b**).

**Table 1:** Demographic and clinical data of all 24 patients.

Age (y)	Uni-**/b**i-lateral	Local Pain +**/**–	Menarche +/–	ALN Enlargement +/–
13.0 (12.0, 14.8)	21/3	3/21	19/5	1/23

**Note: **Age is presented as median (first quantile, third quantile); y: years; ALN: axillary lymph node.

**Table 2:** Grayscale ultrasound features of all lesions.

Parameters	Characteristics	Number of Lesions (%)
Shape	Oval	23 (85.2%)
-	Round	1 (3.7%)
-	Irregular	3 (11.1%)
Orientation	Parallel	26 (96.3%)
Margin	Circumscribed	26 (96.3%)
Lobulated	-	9 (33.3%)
Encapsulated	-	22 (81.5%)
Internal echo pattern	Uniform hypo	16 (59.3%)
-	Hetero hypo	11 (40.7%)
Posterior acoustic features	No features	16 (59.3%)
-	Enhancement	11 (40.7%)
-	Shadowing	0 (0.0%)
Calcification	-	0 (0.0%)
Duct ectasia	-	0 (0.0%)
Skin changes	No changes	15 (55.6%)
-	Thickening	12 (44.4%)

**Note: **Uniform hypo: uniform hypoechogenicity; Hetero hypo: heterogeneous hypoechogenicity.

**Table 3:** Ultrasonic color doppler data of all lesions.

Alder Classification	Number of Lesions (%)
Grade 0	3 (11.1%)
Grade 1	11 (40.7)
Grade 2	7 (25.9%)
Grade 3	6 (22.2%)
RI > 0.7	1 (3.7%)

**Abbreviation: **RI: Resistance index of blood flow.

## Data Availability

The data used in this study is available within the article.

## LIST OF ABBREVIATIONS

US: Ultrasound
RI: Resistance Index
BI-RADS: Breast Imaging Reporting and Data System

## STANDARDS OF REPORTING

STROBE guidelines were followed.
